# Robust data-driven NLMPC for real-time microgrid management under uncertainties and false data injection attacks

**DOI:** 10.1038/s41598-026-48944-y

**Published:** 2026-04-16

**Authors:** Elaheh Yaghoubi, Elnaz Yaghoubi, Mehdi Zareian Jahromi, Mohammad Reza Maghami, Mohamed Mazlan

**Affiliations:** 1 Department of Electrical and Electronics Engineering, Istanbul Topkapi University, Istanbul, Turkey; 2 Department of Electrical and Electronics Engineering, National Kaohsiung University of Science and Technology, Kaohsiung City, Taiwan; 3 Strategic Research Institute (SRI), Asia Pacific University of Technology and Innovation (APU), 57000 Kuala Lumpur, Malaysia; 4 Faculty of Artificial Intelligence and Cyber Security (FAIX), Universiti Teknikal Malaysia Melaka, Malacca, Malaysia

**Keywords:** Microgrid control, Nonlinear model predictive control, Bayesian neural network, False data injection attacks, Uncertainty management, Renewable energy integration, Energy science and technology, Engineering, Mathematics and computing

## Abstract

The integration of renewable energy sources in microgrids (MGs) enhances system efficiency but increases vulnerability to cyberattacks such as false data injection (FDI) attacks. This paper presents a robust data-driven nonlinear model predictive control (NLMPC) framework with the integration with Bayesian Neural Networks (BNNs). The BNN offers probabilistic state estimation, allowing uncertainty-aware prediction and early anomaly detection. By integration BNN within the NLMPC, the framework obtains combined detection, mitigation, and control of cyberattacks. Simulation results show that the proposed framework detects FDI attacks within 0.1 s, and stability is restored within 0.4 s. Frequency deviation is reduced by 99.7%, while active and reactive power fluctuation decreases by 85% and 87%, respectively. Moreover, battery storage operation remains within a safe limit. These results validate the effectiveness of the proposed framework for real-time cyber-resilient microgrids control under uncertainty and attack scenarios.

## Introduction

### Importance and motivation

The use of distributed renewables in microgrids (MGs) enhances sustainability, but increases the variability due to fluctuations in solar, wind, and battery energy storage systems (BESS) generation[1–3]. At the same time, the growing dependence on advanced measurement and communication infrastructure exposes MGs to cybersecurity risks. Among various types of cyber-attacks, false data injection (FDI) attacks are particularly dangerous because they can manipulate sensor measurements and deceive the control system without triggering traditional anomaly detection mechanisms. As a result, real-time operation of MGs now demands intelligent and robust control systems that can not only manage uncertainty but also adapt to cyber-related disturbances. This need is especially critical when the MG operates in grid-connected mode, as faulty measurements or poor control commands can spread instability to the main grid[4]. The novelty of this research lies in combination of probabilistic learning and real-time predictive control in a unified framework, allowing joint uncertainty assessment, cyberattacks detection, and adaptive microgrid control. In order to overcome the conventional control limitations and improve the cyber-physical resilience of microgrids, a unified BNN-based NLMPC framework is proposed in this study. The main contributions of this study can be summarized as follows:

- A novel uncertainty-aware control architecture that integrates BNNs into real-time NLMPC, enabling reliable decision-making under nonlinear dynamics, stochastic renewable generation, and measurement uncertainty.
- A probabilistic dynamic learning approach, where the BNN infers both state predictions and their epistemic uncertainty, allowing early identification of abnormal system behavior including false-data-injection (FDI) attacks.
- A comprehensive robustness analysis using Monte Carlo simulations to evaluate the controller’s performance under renewable variability, load disturbances, and cyberattack scenarios.
- Demonstration of significant performance gains over traditional deterministic controllers in terms of dynamic stability, operating cost, and resilience against cyberattacks, validated through a suite of realistic microgrid simulations.

### Literature review

The integration of renewables in cyber-physical MGs demands control approaches robust to uncertainty and cyberattacks. Nonlinear model predictive control (NLMPC) is widely used for real-time constrained control [2, 5, 6]. Data-driven modeling overcomes white box models, which are error-prone and require detailed system knowledge. For instance, as reported in [7], an artificial neural network (ANN)-based BESS controller improved damping of inter-area oscillations compared to traditional stabilizers. However, it lacks real-time cyber resilience (no detection or mitigation mechanism) and does not handle the modeling challenges in hybrid microgrids incorporating both rotating and non-rotating sources of renewables. Study [8] proposes a support vector regression (SVR)-based interval power flow method for distributed generations (DGs) integrated distribution networks, enhancing efficiency and uncertainty management. However, it lacks real-time cyber-resilience, probabilistic uncertainty assessment, and hybrid MG modeling. Study [9] has proposed a gaussian process regression (GPR)-based method that can be used to effectively handle uncertainties and dependencies in net load errors in microgrids, outperforming copula-based models. However, this study does not cover cyber resiliency in real-time or hybrid microgrid models with mixed sources. A Bayesian regularized Deep Neural Network (BDNN)-based strategy [10] is proposed for BESS control of DC MGs, achieving accurate SoC management and enhanced reliability, as validated by simulation and hardware-in-the-loop (HIL) tests. However, this approach does not address real-time resilience to cyberattack and hybrid MG modeling challenges. Cybersecurity studies mainly focus on FDI attacks, which can increase smart grid operating costs [11]. Traditional bi-level linear programming is impractical for large systems and real-time operation, highlighting the requirement for dynamic methods. In addition, the study in [12] indicates that static approaches are unable to identify intra-interval dynamics when assessing FDI vulnerabilities, which are underestimated in the presence of variable resources. However, a dynamic model is able to identify these risks. In study [13], a DRO-based strategy is proposed for addressing FDI load redistribution attacks under uncertainty in renewable energy sources, enhancing efficiency and security compared to worst-case methods. However, it does not consider real-time response or intra-interval dynamics. In reference [14] authors investigate FDI attack in networked control systems and present algorithms to create undetectable attacks for two systems models, along with conditions under which attacks can bypass a χ^2^ failure detector. The study focuses on attack construction and planning rather than real-time detection or on-line control updates. Another paper [15] introduces a controller that detects and reduces FDI attacks using Kalman-based estimation and neural anomaly detection, demonstrating strong robustness. Study [16] presents an optimal FDI intrusion detector for time-varying smart grids, enabling fast, stable detection with controlled false alarms. Another research [17] proposes an adaptive nonparametric cumulative sum (AN-CUSUM) detector for online detection of coordinated cyber-physical and FDI attacks, achieving improved detection and isolation performance over standard cumulative sum methods.

Recent studies have addressed cyber resilience in BESS using advanced data-driven techniques. For example, Rafy et al. [18] proposed a beta-VAE-based anomaly detection model for IoT-enabled BESS. On the other hand, the proposed BNN-based framework allows clear probabilistic state estimation, and integrates uncertainty-aware predictions within the NLMPC, allowing combined detection, mitigation, and real-time control adaptation. Moreover, IoT-based energy management using approaches [19] mainly deals with monitoring, scheduling, and system coordination without considering real-time cyberattack the mitigating within closed-loop control. This shows the novelty of the proposed approach.

In order to better define the proposed framework with respect to the closest existing approaches, it is necessary to mention that ANN-based and traditional data-driven NLMPC methods mainly enhance prediction and control performance but remain deterministic and do not quantify predictive uncertainties. Similarly, methods based on SVR and GPR enhance uncertainty representation for forecasting or operational studies, but they lack an integrated framework for real-time FDI detection and closed-loop resilient control. Moreover, existing cyberattacks-oriented studies typically focus on detection alone, or an attack modeling and assessment of vulnerabilities, without integrating the probabilistic learning and online control reconfiguring into a unified predictive control framework. Therefore, the main novelty of this research is not the isolated use of BNN or NLMPC, but their tight integration for simultaneous uncertainty-aware prediction, anomaly detection, attack mitigation, and real-time control adaptation in a hybrid grid-connected microgrid.

There are not many studies that deal with real-time FDI attacks, particularly in grid-connected microgrids, where false data can undermine the stability of both local and main power systems. The combination of BNNs and NLMPC for real-time FDI mitigation has not yet been widely proposed. All data-oriented microgrid controllers are deterministic; therefore, they lack uncertainty awareness, which makes them unable to detect anomalies caused by cyberattacks. Uncertainty-aware predictions within predictive control, achieved through BNN, allow adaptive responses to abnormal behavior. Despite these developments, existing methods are unable to offer a unified framework that deals with nonlinear system dynamics, uncertainty quantification, and real-time resiliency against cyberattacks in grid-connected microgrids.

- **Limited real-time resilience to cyberattacks** Existing studies largely address cyber threats at the design or planning stage, with few methods capable of real-time attack detection and active recovery during operation.
- **Absence of uncertainty quantification** Most data-driven models do not assess prediction uncertainty, hindering their ability to detect FDI attacks or support risk-aware decision-making.
- **Rare integration of probabilistic models into NLMPC** Although BNNs and model predictive control have been studied independently, their combined use for real-time resilient microgrid control is largely unexplored.
- **Modeling limitations in hybrid microgrids** Accurately representing the coupled dynamics of rotating and non-rotating distributed energy resources remains a substantial challenge for both first principles and purely data-driven approaches.

This research is organized as follows: “Proposed method architecture” section reviews MG control, data-driven modeling, and cybersecurity. “System modeling and component dynamics” section introduces the suggested BNN-based NLMPC framework. “Simulation and results” section details the simulation setup, attack scenarios. “Conclusion” section concludes the paper, and “Future directions” section presents the directions for future studies.

## Proposed method architecture

**Fig. 1:**
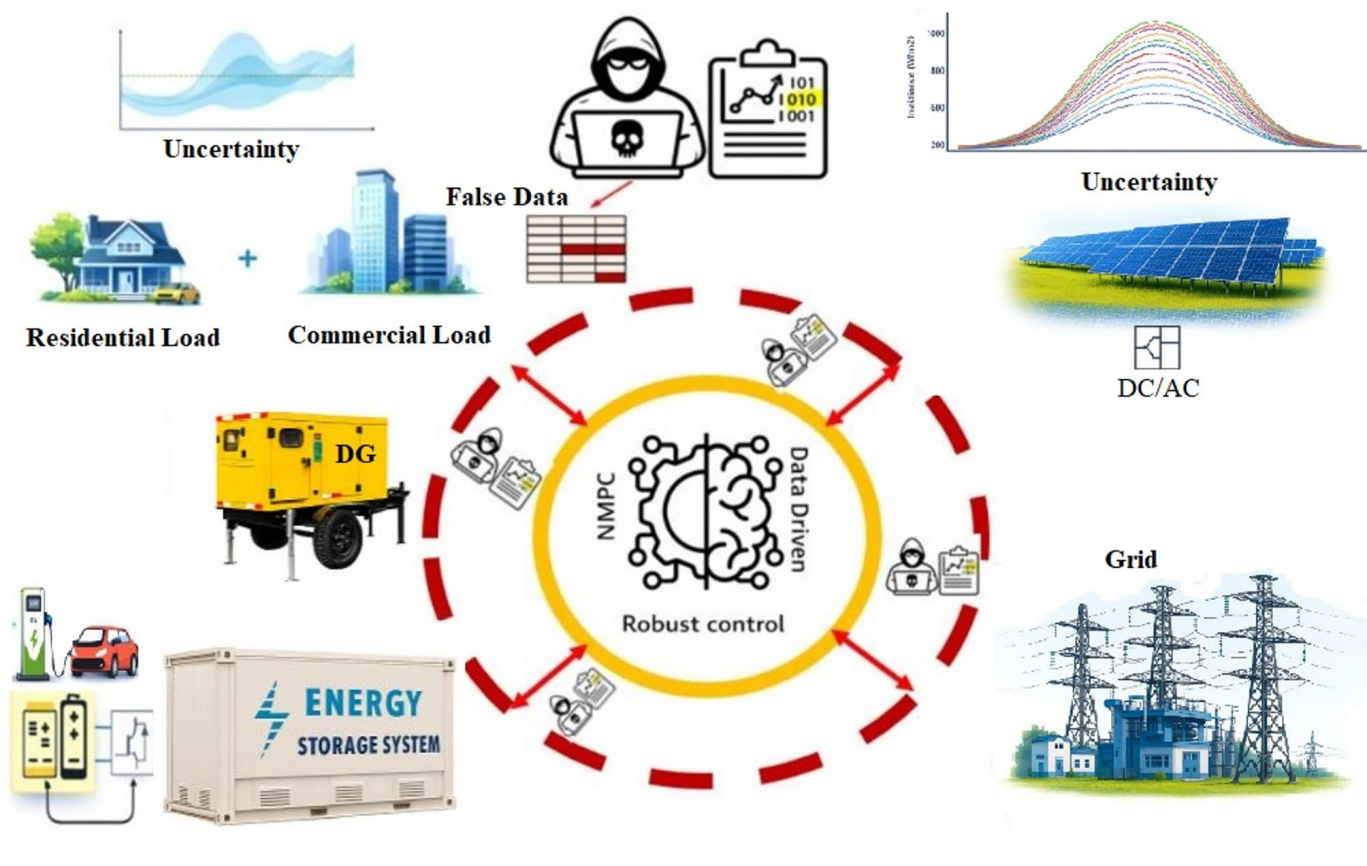
Conceptual Proposed strategies in grid-connected MG.

The suggested control framework enables robust, real-time operation of a grid-connected MG under the renewable uncertainty and FDI attacks, using a BNN-based data-driven NLMPC for making uncertainty-aware, accurate decisions. Figure 1 illustrates the MG architecture and the robust data-driven NLMPC controller. The MG consists of a utility, PV panels, a diesel generator (DiG), a BESS, and three inverters, each with a specific grid-support function:

- Inverter 1: Shared with the utility grid, acting as the grid-forming.
- Inverter 2: Connected in parallel with the PV arrays, serving as a grid coordinated.
- Inverter 3: Paired with the BESS, operating as a grid-following during charging, and switching to grid-coordinated during discharging.
- DiG: Connected directly to the MG without an inverter, capable of fast response and providing additional stability under transient conditions.

In this setup, the robust data-driven NLMPC coordinates the inverter and DiG set-points to reduce FDI attacks, which manipulate measurements or signals and can cause mismatches, deviations, and instability. By combining a BNN, the NLMPC models uncertainty, rejects corrupted measurements, and computes robust set-points to maintain frequency stability. A state-space BESS model is used in the real-time NLMPC to drive microgrid outputs to references (control inputs $\mathcal{u}(t)$ and outputs $\mathcal{Y}(t)$), while BNN-based predictions offer uncertainty estimates to increase resilience to FDI attacks and disturbances.

1 $$\mathcal{J}={min }_{{\{\mathcal{u}}_{t}\} }{\mathbb{E}}_{\zeta }[{\int }_{{t}_{0}}^{{t}_{0}+T}\left({\left(\mathcal{Y}\left(t\right)-{\mathcal{Y}}_{ref}\left(t\right)\right)}^{T}\mathfrak{Q}\left(\mathcal{Y}\left(t\right)-{\mathcal{Y}}_{ref}\left(t\right)\right)+{\dot{\mathcal{u}\left(t\right)}}^{T}\mathfrak{R}\dot{\mathcal{u}\left(t\right)}+{ \mathcal{u}\left(t\right)}^{T}S\mathcal{u}\left(t\right)+penalt{y}_{FDI}(\dots )\right)dt$$

The system output vector at time $t$ (such as, voltages, frequencies, or power) is denoted by ${\mathcal{Y}}_{t}$, while ${\mathcal{Y}}_{ref}$ represents the desired reference trajectory. The first of the three penalties, $\mathfrak{Q}$ addresses tracking errors; $\mathfrak{R}$ penalizes control input variations, and $S$ penalizes input magnitude, representing operational costs. ${\mathbb{E}}_{\zeta }$ captures uncertainties, noise, or disturbances, enhancing system robustness. Additionally, an optional penalty, $penalt{y}_{FDI}$ imposes stricter restrictions during FDI attacks, improving cyber-resilience while discouraging aggressive control actions. According to Eq. (2), increases in control input are penalized to prevent overly aggressive control inputs.

2 $$\dot{u\left(t\right)}=\frac{d}{dt} \mathcal{u}(t)$$

At each sample instant, the NLMPC performs three main tasks:

1. It predicts future system outputs and states using current measurements and the BNN model.
2. It solves a constrained optimization problem to find the best trade-off between economic and technical objectives.
3. It applied only the first control action from the optimized sequence, then repeats the process at the next step (the receding horizon principle).

This structure ensures efficient, reliable, and robust MG operation, even in the presence of uncertainties and potential data corruption from FDI attacks.

**Fig. 2:**
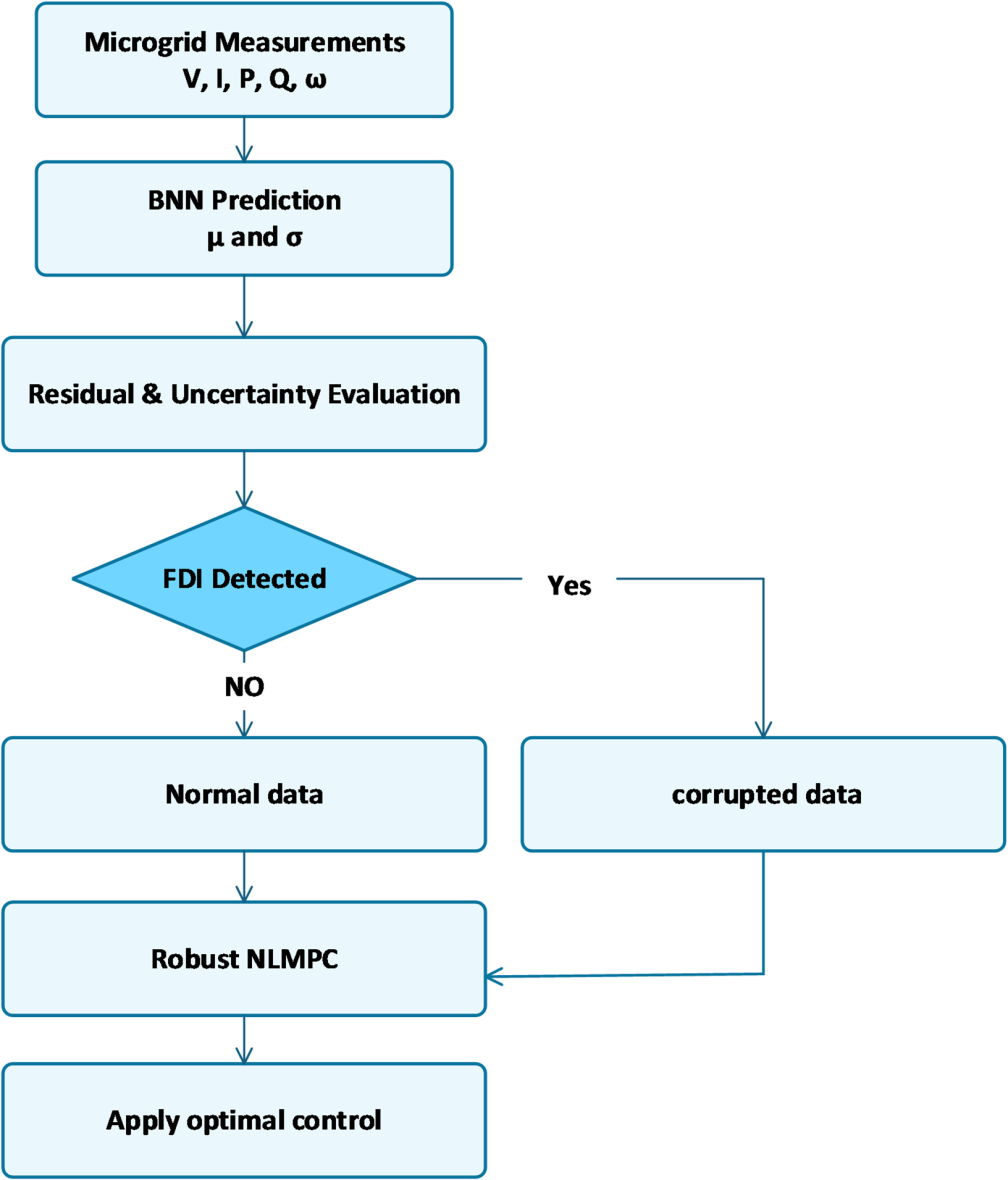
Flowchart of the proposed BNN-based NLMPC framework, illustrating state prediction, uncertainty estimation, FDI detection, and control re-optimization.

The overall structure of the proposed BNN-based NLMPC framework is depicted in Fig. 2, showing that it includes probabilistic state estimation, anomaly detection, and control reconfiguration in a closed-loop system.

### Framework overview

Data-driven strategies use real-time system measurements to identify and model system dynamics without requiring detailed physical models. In the robust data-driven NLMPC framework, system measurements are used to model dynamics and predict the future behaviour of DGs. This enables real-time and reliable MG operation, even under uncertainties and cyberattacks, particularly FDI attacks. Unlike traditional dynamic models that need exact physical parameters (Eq. (3)), the system dynamics in this study are modeled using a BNN (shown in Eq. (4)).

3 $$\dot{X}(t)=\mathcal{A}X(t)+\mathcal{u}\left(t\right)+\mathcal{w}\left(t\right)$$

where, $\mathcal{A}$ and $\mathcal{B}$ are system matrices from physical modeling, and $\mathcal{w}\left(t\right)$ represents modeling error and process noise.

4 $$\dot{X}(t)={\mathfrak{E}}_{BNN}(X(t)+\mathcal{u}\left(t\right))+\mathcal{w}\left(t\right)$$

where ${\mathfrak{E}}_{BNN}\left(X\left(t\right)+\mathcal{u}\left(t\right)\right)$ is the nonlinear mapping learned from data using a BNN, which provides the mean prediction and the associated uncertainty of each state estimate. BNN learns nonlinear dynamics and uncertainties from historical and real-time data, enabling accurate system response prediction. During FDI attacks, the BNN identifies abnormal behavior in tampered measurements or control signals and reduces their influence on control decisions. The integrated framework system effectively manages system uncertainties and offers protection against corruption measurement. By incorporating uncertainty with Monte Carlo simulations, the controller improves stability and situation awareness, allowing stable and secure microgrid operation under uncertainty and FDI attacks. The suggested method is summarized in Algorithm 1. In order to improve the clarity of this framework, the system architecture is presented in this section, and BNN training procedures are explained in “System modeling and component dynamics”.

**Algorithm 1:**
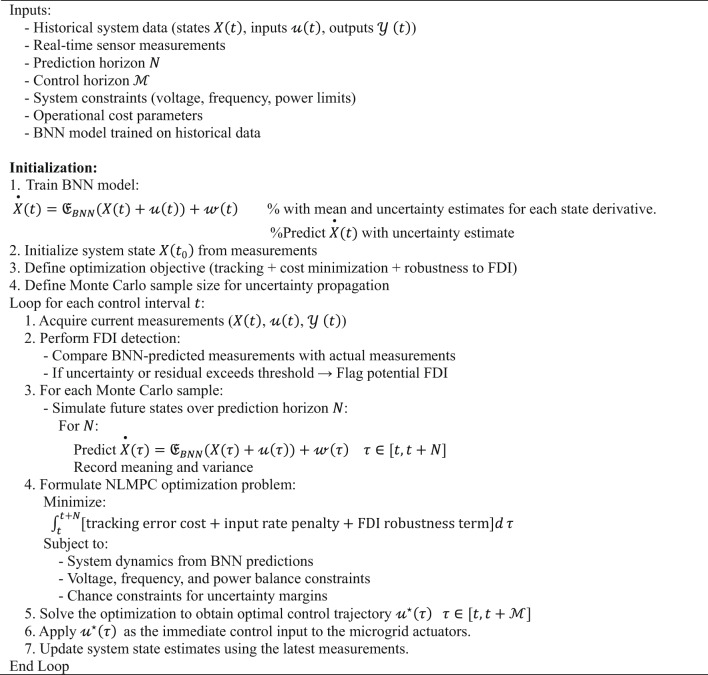
Robust data-driven NLMPC for MG operation under FDI attacks.

## System modeling and component dynamics

This part presents the control objective, constraints, MG dynamic models, and main performance metrics, such as cost, losses, stability, voltage deviation, and storage, used in the NLMPC. Real-time stability assessment is critical, but frequent topology changes challenge traditional models. To overcome this issue, network-preserving models (NPMs) are utilized instead of reduction techniques, maintaining full topology and components (DGs, loads, DiG, and BESS). This structure-preserving approach represents MG nonlinear dynamics and integrates with data-driven predictors. NPMs use sparse matrices and specialized solvers for efficient, fast computation. The next sections introduce the component models forming the predictive dynamics of the suggested approach.

### BNN modeling

A BNN predicts states and uncertainties within the NLMPC to detect FDI attacks, trained on 100,000 Monte Carlo samples (“Modeling of cost”). The Monte Carlo dataset used $1000\times 100$ samples (864 s interval). The BNN model is trained offline using Monte Carlo datasets, and the trained model is deployed in the NLMPC framework for online operation. During online operation, the network parameter values remain constant, and only forward propagation is performed, ensuring computational efficiency. The NLMPC runs in real-time with a 0.01 s step. To enhance cyber-robustness, 20% of the data consisted of ± 15% FDI modification. The BNN has three 64-neuron ReLU layers, with weights learned via Variational inference and hyperparameters tuned by grid search. The BNN was trained with Adam (learning rate 0.001, batch size 64, 20% validation), achieving $MSE< 0.01$. Coarse data Δt = 864 s data is interpolated for 0.01 s NLMPC predictions, with noise and uncertainties modeled as zero-mean Gaussian:

5 $${\mathfrak{Z}}_{t}\sim N(0,\sum t)$$

**Fig. 3:**
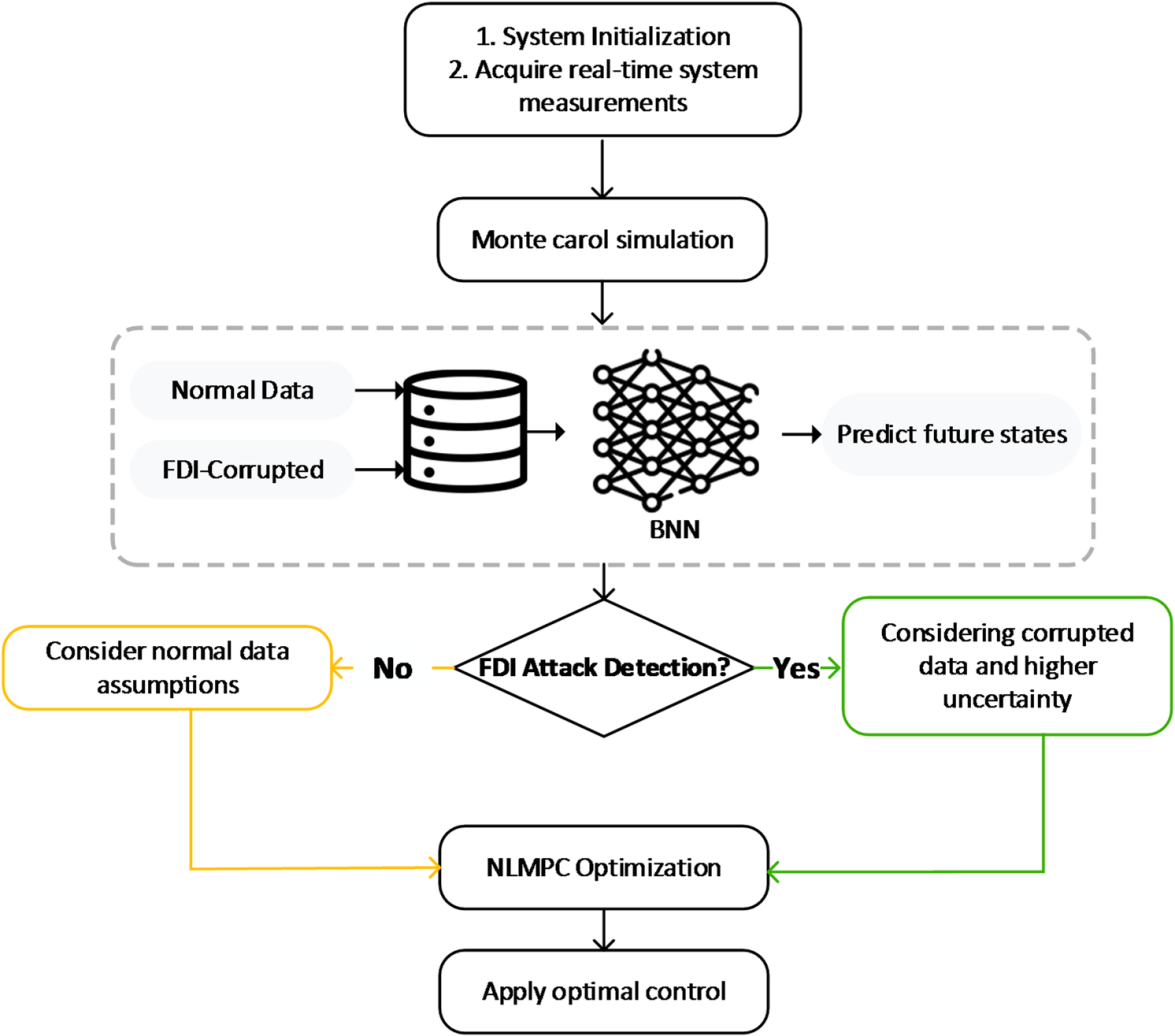
flowchart of robust data-driven NLMPC with BNN for secure MG control.

Covariance matrix $\sum t$ captures uncertainty magnitude and correlation (Eq. (4)). BNN mean (μ) and variance (σ^2^) guide the NLMPC for low-uncertainty, FDI attacks, and risk-aware decisions (Fig. 3, “Detection and impact of cyberattacks” section).

### LC filtered inverter model for data-driven NLMPC

**Fig. 4:**
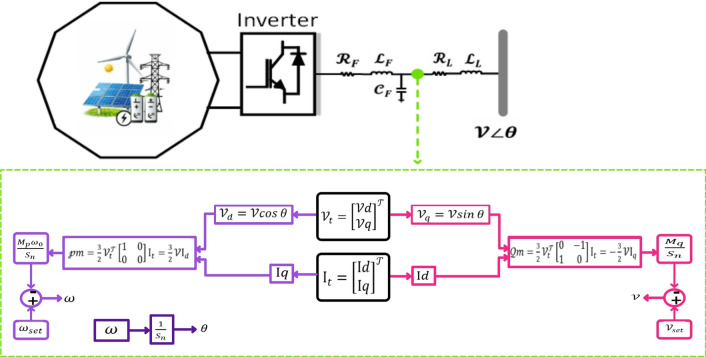
Inverter model with LC filter in the robust data-driven MPC framework.

Figure 4 presents the fifth-order dq-frame inverter with an LC filter integrated into the robust data-driven NLMPC for nonlinear performance optimization.

From Fig. 4, the phase angle is given by:

7 $$\theta =\omega -{\omega }_{0}$$

The terminal voltage vector of the inverter ${\mathcal{V}}_{t}\in {\mathbb{R}}^{2}$ and output current vector ${{\rm I}}_{t}\in {\mathbb{R}}^{2}$ are as follows:

8 $${\mathcal{V}}_{t} = {\mathcal{V}}\left[ {\begin{array}{*{20}c} {\cos \theta } \\ {\sin \theta } \\ \end{array} } \right],I_{t} = \left[ {\begin{array}{*{20}c} {Id} \\ {Iq} \\ \end{array} } \right]$$

where $\mathcal{V}$ and $\theta$ represent the amplitude and phase angle of the inverter terminal voltage after passing through the LC filter, respectively. The dynamics of frequency deviation, voltage, and current are given in state-space form as:

9 $$\varsigma \frac{d\omega }{dt}={\omega }_{set}-\omega -\frac{{M}_{p}{\omega }_{0}}{{S}_{n}}\mathcal{p}m$$

10 $$\varsigma \frac{d\mathcal{V}}{dt}={\mathcal{V}}_{set}-\mathcal{V}-\frac{{M}_{q}}{{S}_{n}}\mathcal{Q}m$$

11 $${\mathcal{L}}_{\mathcal{T}}\frac{d{I}_{t}}{dt}={\mathcal{V}}_{t}-{\mathcal{V}}_{0}\mathfrak{F}-{\mathcal{R}}_{\mathcal{T}}{{\rm I}}_{t}+{\omega }_{0}{\mathcal{L}}_{\mathcal{T}}\mathbf{J}{{\rm I}}_{t}$$

The ${\omega }_{0}\in {\mathbb{R}}$ is nominal grid angular frequency (Hz), and nominal bus voltage magnitude is shown by ${\mathcal{V}}_{0}\in {\mathbb{R}}$ (Volts). The time constant of the low-pass filter in the frequency and voltage regulation loops is represented as $\varsigma \in {\mathbb{R}}^{+}$ (seconds). The droop gains of frequency and voltage ${M}_{p},{M}_{q}\in {\mathbb{R}}^{+}$, respectively. Additionally, the rated inverter apparent power is ${S}_{n}\in {\mathbb{R}}^{+}$ (VA). ${{\omega }_{set},\mathcal{V}}_{set}\in {\mathbb{R}}$ are frequency and voltage setpoints received as control references. The 90° rotation matrix in dq-frame is $$\mathbf{J}=\left[\begin{array}{cc}0& -1\\ 1& 0\end{array}\right]$$, and $$\mathfrak{F}=\left[\begin{array}{c}1\\ 1\end{array}\right]$$ is used for power calculations.

Terminal resistance and inductance are given by Eqs. (12) and (13):

12 $${\mathcal{R}}_{\mathcal{T}}={\mathcal{R}}_{F}+{\mathcal{R}}_{L}$$

13 $${\mathcal{L}}_{\mathcal{T}}={\mathcal{L}}_{F}+{\mathcal{L}}_{L}$$

Here, ${\mathcal{R}}_{\mathcal{T}}$ and ${\mathcal{L}}_{\mathcal{T}}$ are the total resistance and inductance between the inverter output and the point of common coupling (PCC). The filter resistance and filter inductance are donated by ${\mathcal{R}}_{F}$ and ${\mathcal{L}}_{F}$, respectively. The line resistance and inductance are ${\mathcal{R}}_{L}$ and ${\mathcal{L}}_{L}$. The real-time active and reactive power outputs, $\mathcal{p}m$ and $\mathcal{Q}m$, are expressed as combined functions of the inverter current and voltage:

14 $$\mathcal{p}m=\frac{3}{2}{\mathcal{V}}_{t}^{\mathcal{T}}\left[\begin{array}{cc}1& 0\\ 0& 0\end{array}\right]{{\rm I}}_{t}=\frac{3}{2}\mathcal{V}{{\rm I}}_{d}$$

15 $$\mathcal{Q}m=\frac{3}{2}{\mathcal{V}}_{t}^{\mathcal{T}}\left[\begin{array}{cc}0& -1\\ 1& 0\end{array}\right]{{\rm I}}_{t}=-\frac{3}{2}\mathcal{V}{{\rm I}}_{q}$$

These equations reflect inverter dq-frame dynamics and integrate with BNN-based NLMPC for resilient microgrid operation.

### Modeling of BESS

The BESS can be represented by its state of charge (SoC), efficiency, and dynamic behavior. Therefore, the power is controlled by charging or discharging:

16 $$\frac{{P}_{\mathfrak{B}ESS}(t)}{{E}_{\mathfrak{B}ESS}}=\frac{d\mathcal{S}oC(t)}{dt}$$

17 $${P}_{\mathfrak{B}ESS-Charge}(t)={\eta }_{Charge}.{\mathcal{I}}_{\mathfrak{B}ESS}(t).{V}_{\mathfrak{B}ESS}(t)$$

18 $${P}_{\mathfrak{B}ESS-DisCharge}(t)={\eta }_{DisCharge}.{\mathcal{I}}_{\mathfrak{B}Ess}(t).{V}_{\mathfrak{B}ESS}(t)$$

where, ${P}_{\mathfrak{B}ESS}\left(t\right)$ is the power flowing into or out of BESS at time $t$. ${E}_{\mathfrak{B}ESS}$ is the total battery energy capacity, representing the maximum energy the battery can store. ${\mathcal{I}}_{\mathfrak{B}ESS}(t)$ is the BESS current (positive during discharging, negative during charging), and ${V}_{\mathfrak{B}ESS}\left(t\right)$ is the BESS terminal voltage. ${\eta }_{Charge} ,{\eta }_{DisCharge}$ are charging and discharging efficiencies, respectively. A BNN models the BESS input–output power to represent nonlinear behaviour under uncertainty and FDI attacks.

20 $${P}_{\mathfrak{B}ESS}={\mathfrak{E}}_{BNN}({\mathcal{I}}_{\mathfrak{B}ESS}\left(t\right),{V}_{\mathfrak{B}ESS}\left(t\right),\mathcal{S}oC\left(t\right))+{\mathfrak{Z}}_{\mathfrak{B}ESS}(t)$$

where, ${\mathfrak{E}}_{BNN}({\mathcal{I}}_{\mathfrak{B}ESS}\left(t\right),{V}_{\mathfrak{B}ESS}\left(t\right),\mathcal{S}oC\left(t\right))$ presents the nonlinear mapping learned by the BNN, and ${\mathfrak{Z}}_{\mathfrak{B}ESS}(t)$ donates the Gaussian noise unrelated to model uncertainty and measurement noise, with ${\mathfrak{Z}}_{\mathfrak{B}ESS}(t)\sim N(0,\sum t)$.

### Modeling of PV panels

Solar modules are typically modeled based on their power output, which depends on ambient conditions such as solar irradiance, temperature, and panel design. The PV array power at time ${P}_{PV}\left(t\right)$ can be expressed as a function of solar irradiance $G\left(t\right)$, cell temperature $T(t)$, and other parameters:

21 $${P}_{PV}\left(t\right)={P}_{STC}.\frac{G\left(t\right)}{{G}_{STC}}.[1+\alpha (T(t)-{T}_{STC})]$$

where, ${P}_{STC}$ is the rated power under standard test conditions (STC). ${G}_{STC}$ and ${T}_{STC}$ are STC irradiance and temperature, respectively, and $\alpha$ is the temperature coefficient of power. To represent nonlinear, time-varying PV power under changing conditions and potential FDI attacks, a BNN model is applied. It predicts the power output and uncertainties from irradiance, temperature, and past PV data, formulated as:

22 $${P}_{PV}\left(t\right)={\mathfrak{E}}_{BNN}(G\left(t\right), T(t))+{\mathfrak{Z}}_{PV}(t)$$

where ${\mathfrak{E}}_{BNN}(G\left(t\right), T(t))$ is the nonlinear mapping learned by the BNN, and ${\mathfrak{Z}}_{PV}(t)$ represents independent Gaussian noise representing model uncertainty and measurement noise, with ${\mathfrak{Z}}_{PV}(t)\sim N(0,\sum t)$.

### Modeling of diesel generator

The diesel generator (DiG) is modeled by its electrical and mechanical dynamics, linking fuel input, rotor speed, and power output. A common simplified model is:

23 $$\mathfrak{l}\frac{d{\varpi }_{DiG}(t)}{dt}={\mathcal{T}}_{\mathcal{m}}-{\mathcal{T}}_{\mathcal{e}}-D({\varpi }_{DiG}(t)-{\varpi }_{0})$$

24 $${P}_{DiG}(t)=K\mathfrak{p}.{u}_{DiG}(t)$$

where $\mathfrak{l}$ is the moment of inertia, ${\varpi }_{DiG}(t)$ is the speed of the rotor. ${\mathcal{T}}_{\mathcal{m}}$ and ${\mathcal{T}}_{\mathcal{e}}$ are the mechanical and electrical torque. $D$ is the damping coefficient, ${\varpi }_{0}$ is the nominal angular speed, $K\mathfrak{p}$ is the power gain, and ${u}_{DiG}(t)$ is the control input, such as fuel valve position. A BNN models the DiG’s nonlinear dynamics under varying loads and FDI attacks, predicting power and response from fuel consumption, pressure, and rotor speed:

25 $${P}_{DiG}(t)={\mathfrak{E}}_{BNN}({u}_{DiG}\left(t\right), {\varpi }_{DiG}(t))+{\mathfrak{Z}}_{DiG}(t)$$

where ${\mathfrak{E}}_{BNN}({u}_{DiG}\left(t\right), {\varpi }_{DiG}(t))$ is the nonlinear mapping of power output learned by BNN from rotor speed and control input, and ${\mathfrak{Z}}_{DiG}(t)$ is given as Gaussian noise. ${\mathfrak{Z}}_{DiG}(t)$ is independent Gaussian noise. ${\mathfrak{Z}}_{DiG}(t) \sim N(0,\sum t)$ denoting model uncertainty and measuring noise.

### Modeling of cost

The cost models for PV panels, BESS, DiG, and the main grid are formulated as follows:

26 $${\mathbf{C}\mathbf{o}\mathbf{s}\mathbf{t}}^{PV}(t)=\frac{{\mathbf{C}\mathbf{o}\mathbf{s}\mathbf{t}}_{capital}^{PV}\times {\mathcal{P}}_{capital}^{PV}\times \mathcal{f}}{{{\rm T}}_{life}\times 8760\times \mathcal{C}{\mathcal{F}}^{PV}}+{\mathbf{C}\mathbf{o}\mathbf{s}\mathbf{t}}_{O\&M}^{PV}\times {\mathcal{P}}^{PV}(t)$$

27 $${\mathbf{C}\mathbf{o}\mathbf{s}\mathbf{t}}^{\mathfrak{B}ESS}(t)=\frac{{\mathbf{C}\mathbf{o}\mathbf{s}\mathbf{t}}_{capital}^{\mathfrak{B}ESS}\times {\mathcal{P}}_{capital}^{\mathfrak{B}ESS}\times \mathcal{f}}{{{\rm T}}_{life}\times 8760\times \mathcal{C}{\mathcal{F}}^{\mathfrak{B}ESS}}+{\mathbf{C}\mathbf{o}\mathbf{s}\mathbf{t}}_{O\&M}^{\mathfrak{B}ESS}\times \left|{\mathcal{P}}^{\mathfrak{B}ESS}(t)\right|\pm {\mathfrak{N}}^{TOU}(t)\times {\mathcal{P}}^{\mathfrak{B}ESS}(t)$$

The cost of PV panel and BESS is represented by a linear equation, while the fixed costs depend on the investment cost, the unit lifetime (${{\rm T}}_{life}$), the annual interest rate ($\mathcal{f}$), and capacity factor ($\mathcal{C}\mathcal{F}$). Variable costs include operating and maintenance costs. Time-of-use pricing for charging/discharging of BESS is represented by ${\mathfrak{N}}^{TOU}$. For the main grid and DiG, the total operating cost is given by Eq. (28):

28 $$\left.\begin{array}{c}{\mathbf{C}\mathbf{o}\mathbf{s}\mathbf{t}}^{\mathfrak{x}}={\mathbf{C}\mathbf{o}\mathbf{s}\mathbf{t}}_{O\&M}^{\mathfrak{x}}(t)+{\mathcal{K}}^{\mathfrak{x}}\times {\mathbf{C}\mathbf{o}\mathbf{s}\mathbf{t}}_{EM}^{\mathfrak{x}}(t)\\ {\mathbf{C}\mathbf{o}\mathbf{s}\mathbf{t}}_{O\&M}^{\mathfrak{x}}(t)=\alpha \times {P}_{\mathfrak{x}}^{2}(t)+\beta \times {P}_{\mathfrak{x}}(t)+\lambda \\ {\mathbf{C}\mathbf{o}\mathbf{s}\mathbf{t}}_{EM}^{\mathfrak{x}}(t)=({C}_{C{O}_{2}}^{\mathfrak{x}}+{C}_{S{O}_{2}}^{\mathfrak{x}}+{C}_{N{O}_{x}}^{\mathfrak{x}})\times {P}_{\mathfrak{x}}(t)\end{array}\right\}\forall \tau \in DiG,Grid$$

29 $${\mathcal{K}}^{\mathfrak{x}}={\left.\frac{{\mathbf{C}\mathbf{o}\mathbf{s}\mathbf{t}}_{O\&M}^{\mathfrak{x}}(t)}{{\mathbf{C}\mathbf{o}\mathbf{s}\mathbf{t}}_{EM}^{\mathfrak{x}}(t)}\right|}_{{P}^{\mathfrak{x}}={P}_{max}^{\mathfrak{x}}}$$

The cost function includes operation and maintenance ${\mathbf{C}\mathbf{o}\mathbf{s}\mathbf{t}}_{O\&M}$ and emissions cost ${\mathbf{C}\mathbf{o}\mathbf{s}\mathbf{t}}_{EM}$. Emissions account for $C{O}_{2}$, $S{O}_{2}$, and $N{O}_{x}$, while the fuel cost of a combined-cycle gas turbine is modeled quadratically as a function of power and cost coefficients (${\boldsymbol{\upalpha}},{\boldsymbol{\upbeta}}, {\boldsymbol{\uplambda}}$). The price penalty coefficient ${\mathcal{K}}^{\mathfrak{x}}$ is provided in Eq. (29).

### Uncertainty using Monte Carlo

Monte Carlo simulation models PV power uncertainties from irradiance, panel performance, and temperature. Solar irradiance $G\sim \mathcal{N}({\mu }_{G},{\sigma }_{G}^{2})$ and temperature $T\sim \mathcal{N}({\mu }_{T},{\sigma }_{T}^{2})$, while load varies uniformly within ± 10%. PV power at each step is calculated as:

30 $${P}_{PV}=G.{{\rm A}}_{PV}.{\mu }_{PV}.{F}_{d}$$

${{\rm A}}_{PV}$ is the PV area, ${\mu }_{PV}$ the efficiency, and ${F}_{d}$ the derating factor. Monte Carlo(1000 iterations,100 100-s intervals) yields PV power statistics converged within ± 1%. The mean and standard deviation are calculated as:

31 $${P}_{PV}^{-}=\frac{1}{N}\sum_{i}^{N}{P}_{PV}^{(i)}$$

32 $${\sigma }_{PV}=\sqrt{\frac{1}{N-1}\sum_{i=1}^{N}{({P}_{PV}^{\left(i\right)}-{P}_{PV}^{-})}^{2}}$$

The 95% confidence interval is determined using the 2.5th and 97.5th percentiles of the output distribution.

33 $${CI}_{95\%}=[{P}_{PV}^{\left(2.5\%\right)},{P}_{PV}^{\left(97.5\%\right)}]$$

A sensitivity analysis is conducted with a non-Gaussian irradiance probability density function (PDF).

### System optimization dynamic control models

The complete dynamic control model for the grid-connected MG includes PV panels, a DiG, BESSs, and three inverter subsystems. As seen from Eqs. (7) to (11), there are five equations per inverter. The MG dynamics are described as a continuous-time nonlinear state-space model for real-time control and optimization, represented by:

34 $$\dot{X}(t)=F\left(X\left(t\right),\mathcal{u}\left(t\right),\mathcal{w}\left(t\right)\right)+\epsilon \left(t\right) \epsilon (t)\sim N(0,\sum t)$$

where $X\left(t\right)\in {\mathbb{R}}^{n}$ is the global state vector at step $t$. $\mathcal{u}\left(t\right)\in {\mathbb{R}}^{m}$ is the control input vector, $\mathcal{w}\left(t\right)$ is an external disturbance such as load fluctuation, solar fluctuation, and FDI attack. The term $\epsilon \left(t\right)$ denotes model uncertainty caused by the BNN integrated into the control structure, with covariance matrix $\sum t$.

#### Inverter subsystem dynamics

Inverters 1 and 2 ($i\in \{\mathrm{1,2}\}$) are modeled as fifth-order nonlinear system:

35 $${X}^{\left(i\right)}\left(t\right)=\left[\begin{array}{c}\begin{array}{c}{\theta }_{i}\left(t\right)\\ {\omega }_{i}\left(t\right)\end{array}\\ {\mathcal{V}}_{i}\left(t\right)\\ {{\rm I}}_{di}\left(t\right)\\ {{\rm I}}_{qi}\left(t\right)\end{array}\right], {\mathcal{u}}^{(i)}(t)=\left[\begin{array}{c}{\omega }_{i}^{set}(t)\\ {\mathcal{V}}_{i}^{set}(t)\end{array}\right]$$

For inverter 3 combined with the BESS, the model includes two additional energy-related states:

36 $${X}^{3}(t)=\left[\begin{array}{c}\begin{array}{c}\begin{array}{c}\begin{array}{c}\begin{array}{c}\begin{array}{c}{\theta }_{3}(t)\\ {\omega }_{3}(t)\end{array}\\ {\mathcal{V}}_{3}(t)\end{array}\\ {{\rm I}}_{d3}(t)\end{array}\\ {{\rm I}}_{q3}\left(t\right)\end{array}\\ \mathcal{S}OC(t)\end{array}\\ {E}_{Stored}(t)\end{array}\right]$$

where $\mathcal{S}OC(t)$ is the SoC of the BESS and ${E}_{Stored}(t)$ is the energy stored.

The DiG is represented by a third-order nonlinear state-space model:

37 $${X}^{\left(DiG\right)}\left(t\right)=\left[\begin{array}{c}\begin{array}{c}{\omega }_{DiG}\left(t\right)\\ {P}_{DiG}\left(t\right)\end{array}\\ {\Gamma }_{DiG}\left(t\right)\end{array}\right], {\mathcal{u}}^{\left(DiG\right)}\left(t\right)={P}_{DiG}^{Set}(t)$$

The dynamics of each MG component $k\in \{\mathrm{1,2},3,DiG\}$ (3 inverters and DiG) are described by:

38 $$\dot{{X}^{(k)}}(t)={F}^{(k)}\left({X}^{(k)}\left(t\right),{\mathcal{u}}^{(k)}\left(t\right),{\mathcal{w}}^{(k)}\left(t\right)\right)+{\epsilon }^{(k)}\left(t\right)$$

where ${\epsilon }^{(k)}\left(t\right)$ is the uncertainty model for component $k$. The dimensions of states and control inputs vary as in Eqs. (35) to (38).

#### Aggregate MG system representation

By combining the states of the individual subsystems, the entire MG system dynamics are expressed as:

39 $$X\left(t\right)=\left[\begin{array}{c}\begin{array}{c}\begin{array}{c}{X}^{\left(1\right)}\left(t\right)\\ {X}^{\left(2\right)}\left(t\right)\end{array}\\ {X}^{\left(3\right)}\left(t\right)\end{array}\\ {X}^{\left(DiG\right)}\left(t\right)\end{array}\right], \mathcal{u}(t)=\left[\begin{array}{c}\begin{array}{c}\begin{array}{c}{\mathcal{u}}^{(1)}(t)\\ {\mathcal{u}}^{(2)}(t)\end{array}\\ {\mathcal{u}}^{(3)}(t)\end{array}\\ {\mathcal{u}}^{(DiG)}(t)\end{array}\right]$$

The system includes 20 coupled nonlinear states: 5 each for inverters 1 and 2 (10 states in total), 7 states for the BESS-coupled inverter, and 3 states for the DiG.

### FDI attack detection and mitigation

In the present study, FDI attacks are modeled as added false signals into the measurement signals used by the controller, including frequency, voltage, and active/reactive power feedback. The false data are represented as a bound random disturbance within $\pm$ 15% of the nominal values, targeting the state variables and the control and monitoring processes. The FDI detection-mitigation scheme compares the measures state $X\left(\mathfrak{t}\right)\in {\mathbb{R}}^{n}$ with the BNN-estimated state ${X}{\prime}\left(\mathfrak{t}\right)$*.* Large deviation shows a potential attack:

40 $$\Vert X\left(\mathfrak{t}\right)- {X}{\prime}\left(\mathfrak{t}\right)\Vert >\vartheta$$

where ϑ is threshold accounting for noise and uncertainty. From Eqs. (1) and (4), the control input $\mathcal{u}\left(\mathfrak{t}\right)$ is obtained by minimizing the cost function subject to system dynamics and chance constraints:

41 $${\mathbb{P}}\left(X\left(t\right), \mathcal{x}\right)\ge 1-\alpha \forall t\in \{{t}_{0},{t}_{0}+T\}$$

The parameter $\alpha$ defines the maximum allowable probability that the system state $X\left(\mathfrak{t}\right)$ exceeds the safety constraints $\mathcal{x}$ within the prediction horizon. Here, $\alpha$ is set to 0.05, meaning there is a 95% confidence that the system remains within the safe area. The main design parameters were chosen based on the dominate fast transient dynamics of the grid-connected microgrid and real-time computational limitations of the proposed control scheme. As the most important inverter- and frequency-driven transients, especially immediately after load disturbances and FDI attack, evolve within the first 0.1–0.2 s, the prediction horizon was selected as ${N }_{p}=20$ steps for 0.01 sample period, corresponding to 0.2 s prediction window. This horizon ais enough to capture the dominate early stages frequency and active and reactive power oscillations that determine the system recovery trend after the attack. The control horizon has been chosen as ${N }_{c}= 5$ steps (0.05 s) in order to reduce the online optimization burden while preserving adequate control actions over the most sensitive part of transient response. The parameters used for the anomaly detection were chosen from the statistical distribution of BNN residual error using the predictive uncertainty bound according to the confidence interval of the form of $\mu \pm 3\sigma .$ This configuration is shown to provide a suitable balance between fast attack detection, low false-positive alarms, and robust operation under stochastic renewable uncertainty. The final parameters were validated by repeated simulation studies to ensure the reported 0.1 s detection time, sub-0.4 s recovery, and compatibility with the 0.01 s real-time control cycle. The detection and mitigation can be described as provided in Algorithm 2.

**Algorithm 2:**
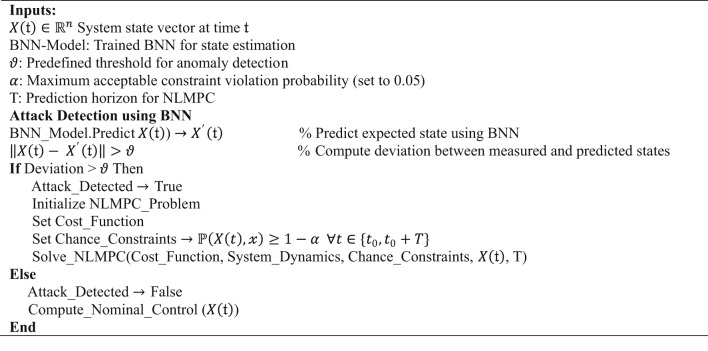
FDI detection and mitigation scheme.

## Simulation and results

### Uncertainty analys parameters

**Fig. 5:**
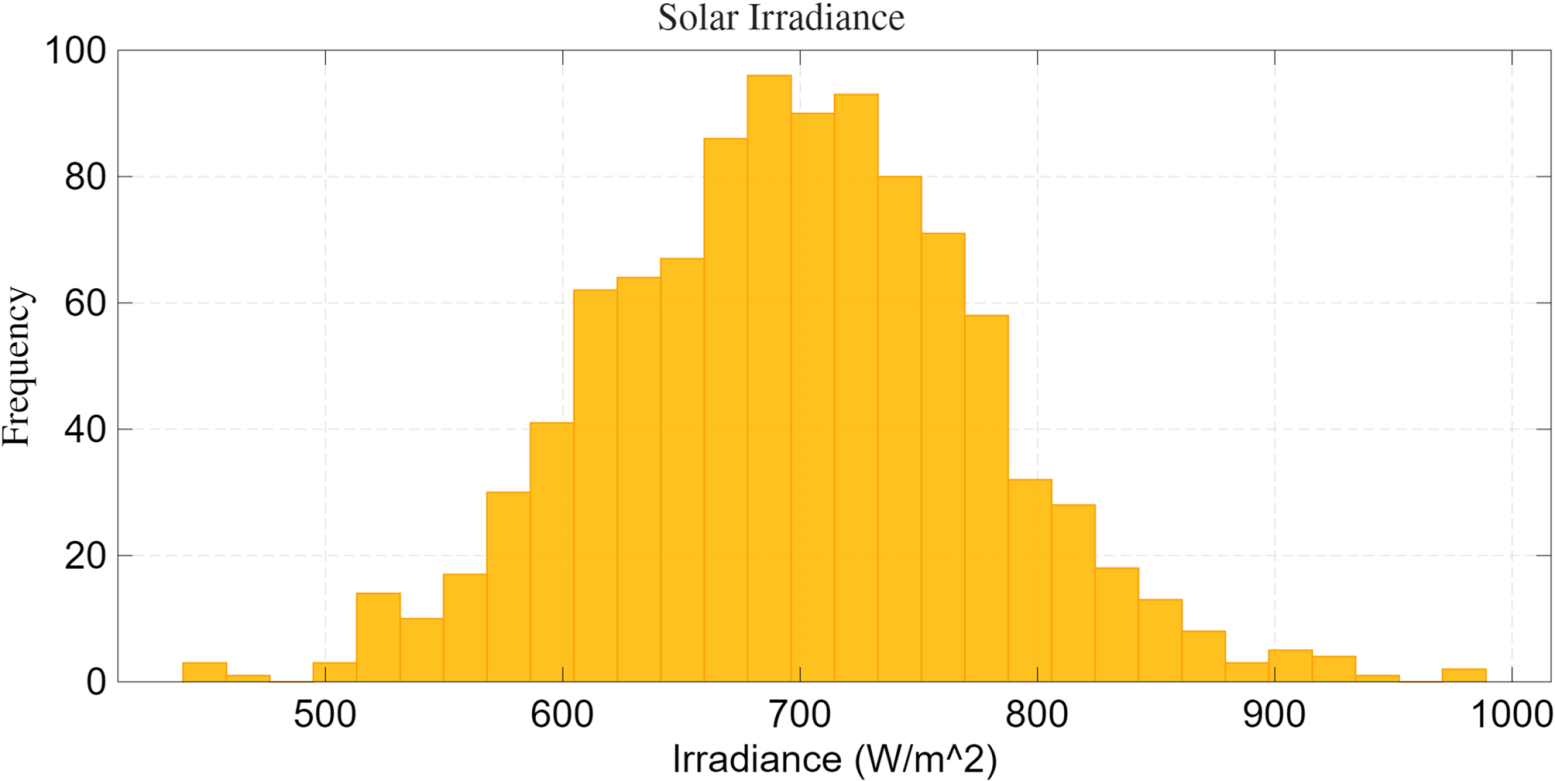
Uncertainty in solar irradiance of PV panels.

**Fig. 6:**
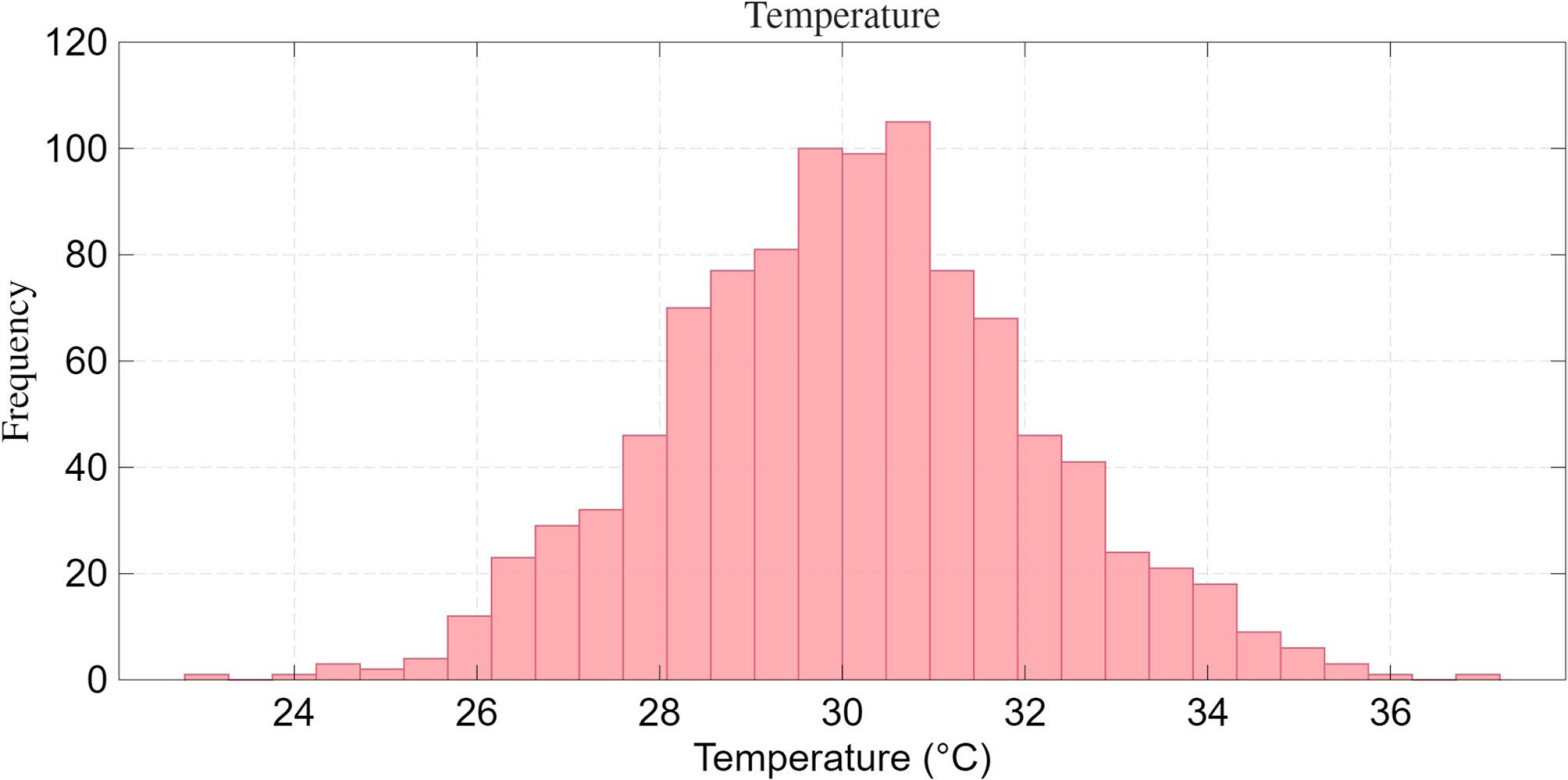
Temperature uncertainty affecting the PV panels.

**Fig. 7:**
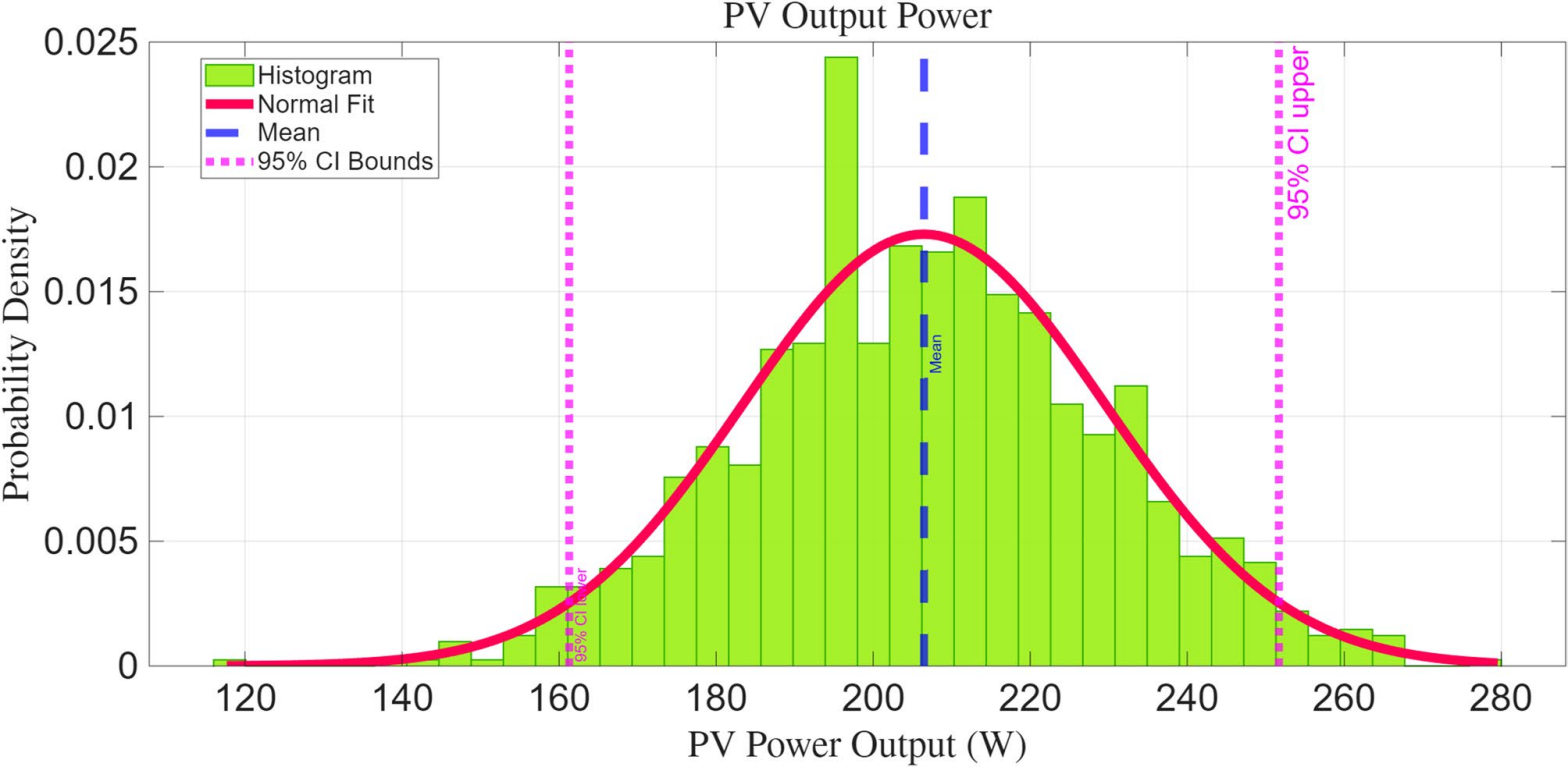
Power output of PV panels under irradiance and temperature uncertainty.

Having established dynamic models and uncertainties, the robust data-driven NLMPC is evaluated through real-time simulations. In the simulation shown in Figs. 5, 6, and 7, the Monte Carlo method is used to model fluctuations in PV irradiance, temperature, and power output.

### Detection and impact of cyberattacks

**Fig. 8:**
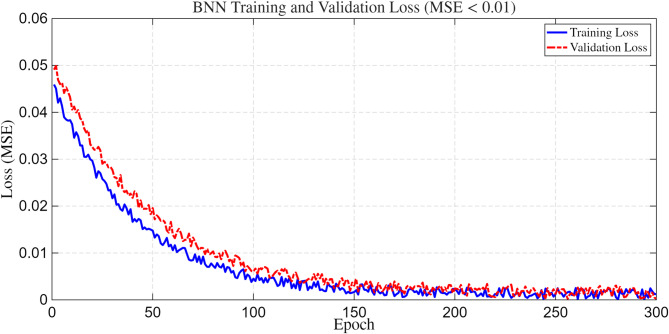
Learning curves of the BNN for MG state prediction.

**Fig. 9:**
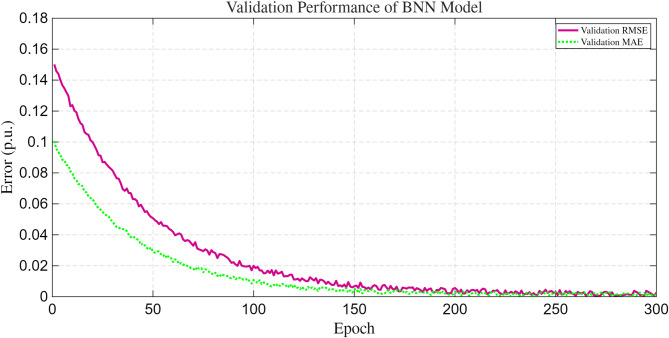
Evolution of RMSE and MAE for BNN state estimation.

The simulation is executed with a control sampling time of 0.01 s to properly capture fast system dynamics. Although the BNN is trained using coarser Monte Carlo data (Δt = 864 s), it is integrated into the controller through interpolation to maintain temporal consistency between prediction and control. For visualization purposes, simulation outputs are recorded every 100 s, while the underlying control loop continues to operate at 0.01 s update rate. Before integration, the BNN model is trained and validated to ensure accurate dynamic state estimation. Figure 8 illustrates the BNN learning curves, with both training and validation losses converging below an MSE = 0.01(p.u.^2^), demonstrating stable learning and proper generalization. Figure 9 illustrates the evolution of RMSE and MAE over the validating data, with final values of RMSE = 0.092 p.u. and MAE = 0.056 p.u., consistent with the predicted accuracy required for the implementation of NLMPC. These results confirm the reliability of the BNN-based state predictor in representing the nonlinear system dynamics under uncertainties.

The performance of the FDI detection mechanism is measured by standard quantitative metrics. The proposed residual-based scheme achieves a detection rate around 98.6%, with a false positive rate below 2%. The average detection time is around 0.1 s, showing rapid response cyberattack conditions. The detection threshold is determined based on the statistical properties of the BNN prediction error and defined using the predictive uncertainty bounds ($\mu + k\sigma$). The value of $k$ is selected to ensure a high confidence level with minimizing false alarms. This uncertainty-aware threshold allows reliable discrimination between normal operating variations and injected false data.

Table 1 indicates BNN performance, showing MSE below 0.01 p.u.^2^ and 2 ms inference time, enabling real-time NLMPC implementation. Low Negative Log-Likelihood (NLL) values ($\sim 1.2$) confirm accurate probabilistic prediction and well-calibrated uncertainty.

**Table 1:** Performance metrics of the BNN model for training and validation.

Dataset	MSE (p.u.^2^)	RMSE (p.u.)	MAE (p.u.)	NLL	Inference time (ms/sample)
Training	8.2 × 10⁻^3^	0.087	0.052	1.18	2.1
Validation	8.9 × 10⁻^3^	0.092	0.056	1.23	2.3
Test	9.4 × 10⁻^3^	0.095	0.058	1.27	2.2

Simulations were conducted on an Intel Core i7 CPU and 16 GB RAM. As indicated in Table 1, the BNN inference time is around 2 ms. Additionally, the total computation time per control cycle, including BNN inference and NLMPC optimization, is around 6–8 ms. This verifies that the proposed framework meets the real-time implementation requirements for a 0.01 s sampling period. Moreover, the proposed method is appropriate for real-time implementation on standard computing systems. For embedded applications, model reduction can be required, but edge computing remains a feasible solution.

System performance is examined in three different scenarios:

- Scenario 1: normal operation with NLMPC.
- Scenario 2: FDI attack without defense.
- Scenario 3: FDI attack with the proposed robust data-driven NLMPC.

**Fig. 10:**
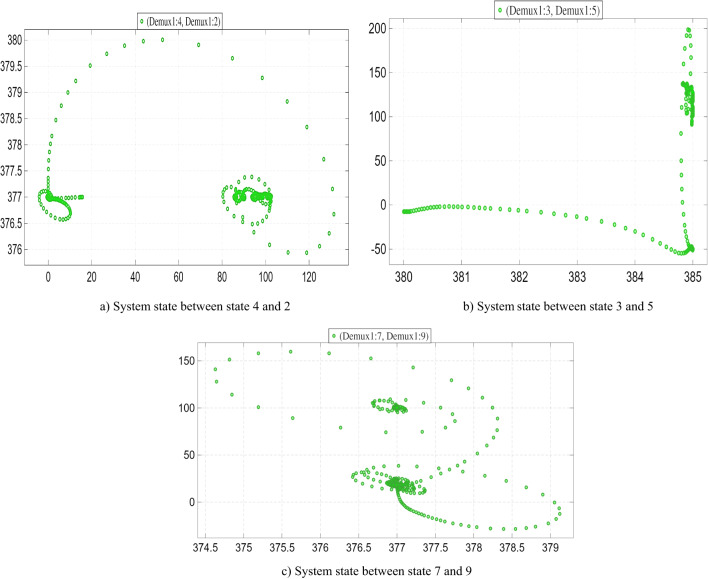
System state responses of MG under normal operating conditions without FDI attack.

In Scenario 1, Fig. 10a–c illustrates system states under normal operation. Without cyberattacks, NLMPC drives states to reference trajectories quickly damping transients from load changes (active power increases from 30 to 140 kW at t = 17 s, and reactive power increases from 20 to 50 kVAR at t = 20 s), ensuring fast convergence and stability.

**Fig. 11:**
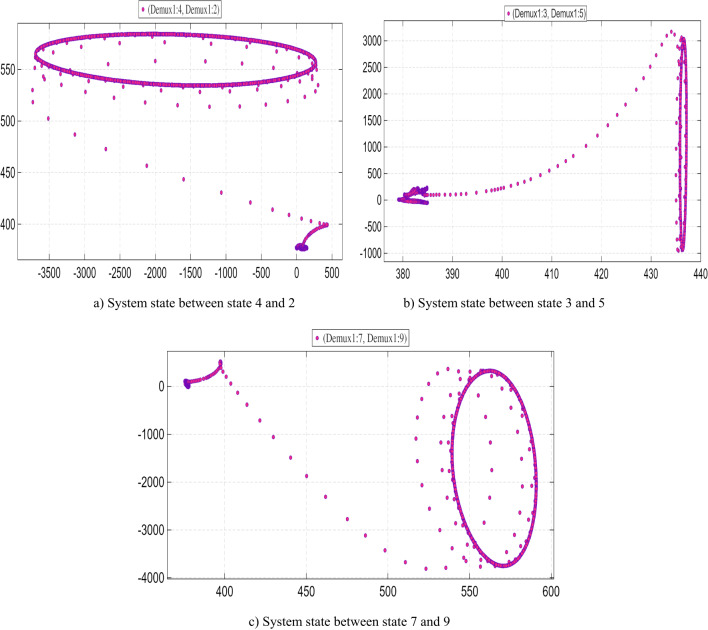
System state responses of MG under normal operating conditions with FDI attack.

At t = 35 s, the FDI attack occurs. The attack magnitude and the time of injection were carefully chosen to represent a realistic challenging cyberattack scenario. The ± 15% An attack level represents a moderate but impactful falsification range, large enough to distort the controller feedback and reveal the vulnerability of deterministic control while still remaining within a plausible measurement manipulation band for stealthy FDI attacks. This attack was initiated at t = 35 s after the system already responded to loads fluctuations at t = 17 s and t = 20 s, therefore, the performance of the controller can be evaluated under both normal transient recovery and a subsequent cyberattack event. This setup provides a clearer assessment of the detection speed, control reconfiguration capability and ability resilient of the proposed framework. For Scenario 2 (Fig. 11a–c), manipulated control signals cause state deviations, large oscillations, and loss of convergence, misleading the controller and disrupting tracking and stability. Scattered data shows that faulty measurements mislead the controller, preventing accurate tracking and steady-state stability.

**Fig. 12:**
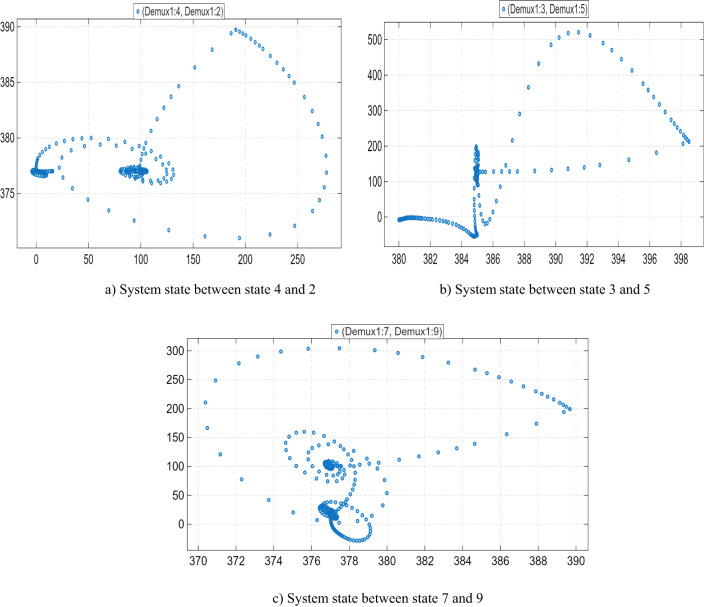
System state responses under FDI attack with proposed robust data-driven NLMPC.

Finally, Scenario 3, which considers the presence of the FDI attack within the proposed robust data-driven NLMPC framework, demonstrates the ability of the system to effectively identify and defend against the impacts of cyberattacks. As shown in Fig. 12a–c, when the FID attacks occur, the proposed BNN-based detection method detects anomalous differences between predicted and measured states. After attack detection, the NLMPC re-optimizes under uncertainty, quickly reducing false data and restoring system stability.

### System performance analysis for different scenarios

**Fig. 13:**
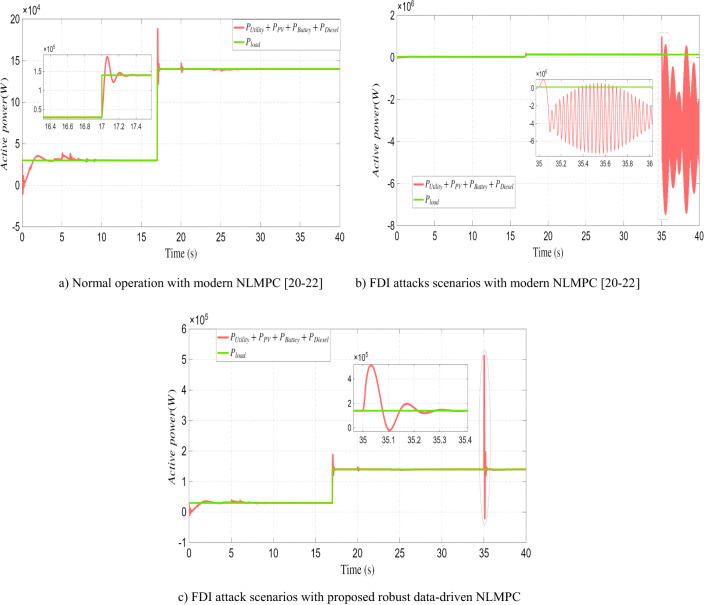
System response under different operating conditions and FDI attack scenarios.

This section evaluates system performance under various operating conditions. Figure 13a–c illustrates responses to varying load and generation profiles. In Scenario 1 (Fig. 13a), before any FDI attack, a sudden increase in active power consumption from 30 to 140 kW at t = 17 s is managed by modern NLMPC, maintaining stability and guiding states to their targets with minor oscillations [20–22]. In Scenario 2 (Fig. 13b), an FDI attack at t = 35 s injected false measurements that mislead the controller, causing large oscillations and loss of synchronism, ultimately destabilizing the network and degrading performance. Scenario 3 (Fig. 13c) illustrates the proposed robust data-driven NLMPC under the same FDI attack. BNN-based detection enables real-time mitigation, restoring stability within 0.4 s with 95% confidence, demonstrating improved robustness over modern NLMPC.

**Fig. 14:**
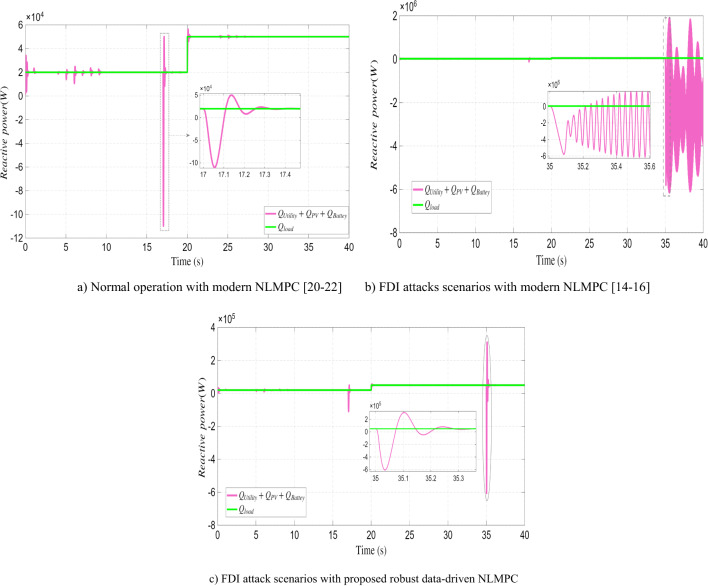
System response under different operating conditions and FDI attack scenarios.

Figure 14a indicates the reactive power of the system under normal conditions. The NLMPC efficiently tracks sudden increases, with reactive power rises from 20 to 50 kVAR at t = 20 s, demonstrating its ability to handle rapid changes [20–22]. At t = 17 s, a sudden active power increase causes a temporary drop. Active power employs all four resources, while reactive power relies on the three inverters. Both stabilize quickly with reactive power oscillations settling within 0.3 s. Figure 14b presents a cyberattack at 35 s. The modern NLMPC maintains stability before attack but fails afterward, with large deviations, demonstrating its inability to handle FDI attacks. Figure 14c shows the proposed robust data-driven NLMPC under the same attack. The controller quickly detects and integrates the FDI impact, with disturbance settling in under 0.3 s, demonstrating superior resilience and stability.

**Fig. 15:**
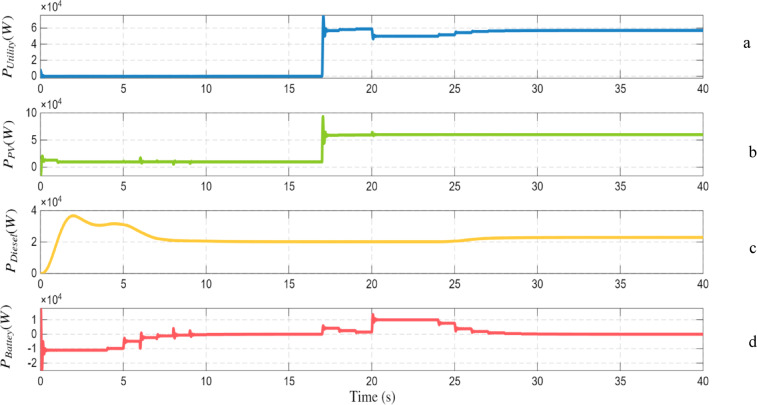
Power sharing behaviour of DERs in normal conditions with moder NLMPC [20–22].

Figure 15 shows DER power-sharing under modern NLMPC, which reacts quickly to sudden power demand changes. In this Figure, PV panels and BESS immediately supply power to the system when it encounters a sudden and huge increase in active and reactive power demand. In the modern NLMPC, DER power distribution follows technical and economic constraints, prioritizing the most efficient resources during high demand [20–22]. PV panels and the BESS are prioritized because of lower generation costs and fast dynamic response, effectively compensating demand fluctuations (Fig. 15b,c). The DiG provides constant active power, enhancing flexibility and supporting stable, efficient power-sharing during load variations (Fig. 15b).

**Fig. 16:**
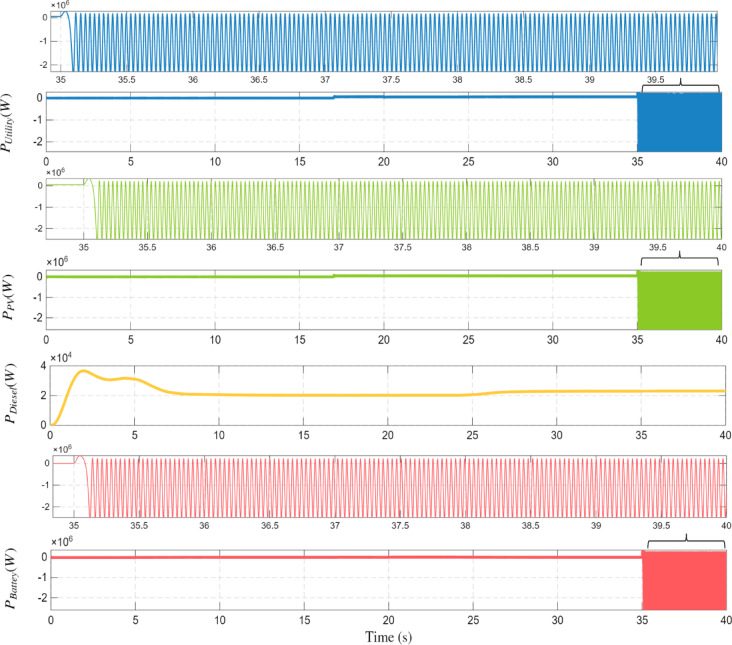
Power sharing behaviour of DERs in FDI attack with modern NLMPC [20–22].

Figure 16 illustrates real-time power sharing among the grid, PV, DiG, and BESS around the FDI attack at t = 35 s. Before the attack, power flows are balanced, and all DERs operate normally. PV and utility supply most active power, while the DiG supports transient, and the BESS smooths fluctuations by charge/discharge control. At t = 35 s, false data corrupts the NLMPC measurement signals. In the magnified regions, modern NLMPC causes sudden oscillations and instability in utility, PV, and BESS outputs. High-frequency oscillations in the utility and PV outputs show the failure of the controller under corrupted data. The DiG covers the shortfall but cannot restore steady state operation, illustrating how the FDI attack disrupts coordinated power-sharing and reduces MG performance.

**Fig. 17:**
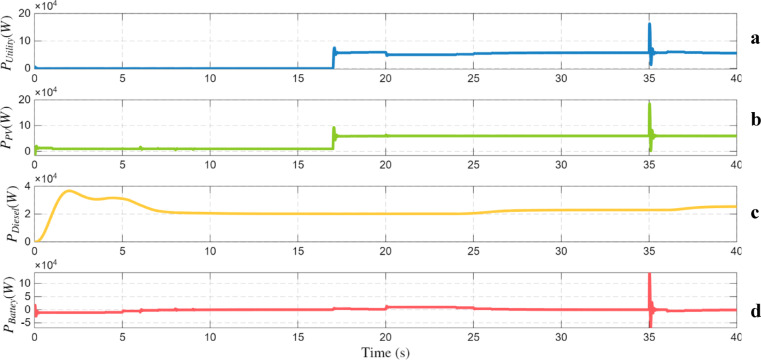
Power sharing behaviour of DERs in FDI attack with proposed robust data-driven NLMPC.

Figure 17 shows the MG power sharing under the suggested framework, during an FDI attack at t = 35 s. Before the attack, the PV and utility units share most of the load, the DiG provides backup, and the BESS handles transients. At t = 35 s, the robust data-driven NLMPC detects deviations between the measured and predicted states and re-optimizes control input in real-time. All power components experience only a brief disturbance, with DER output returning to nominal within 0.4 s, demonstrating the ability of the controller to detect and reduce FDI attacks while maintaining system stability.

**Fig. 18:**
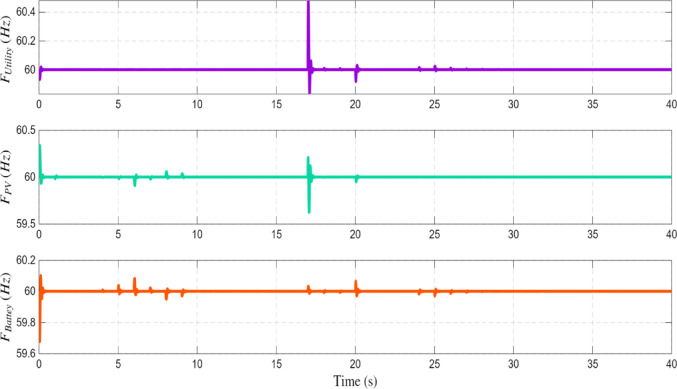
Frequency response of MG under sudden load variation by modern NLMPC [20–22].

Figure 18 shows the frequency fluctuations of the three sources. During steady-state operation, the frequencies of all sources remain close to the nominal frequency of 60 Hz. When a sudden increase in active power demand happens at t = 17 s, transient frequency deviations appear in all sources. However, the modern NLMPC effectively controls the system response, quickly damping the oscillations and restoring the frequencies to their nominal values within a short period. Moreover, it is observed that the PV and BESS exhibit smaller frequency deviations than the utility grid, indicating their effective contribution to maintaining network stability and enhancing overall frequency resilience.

**Fig. 19:**
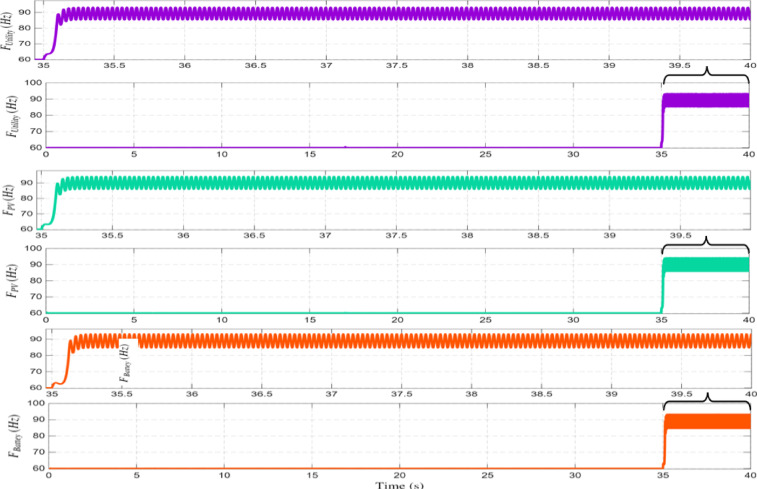
Frequency response of MG under FDI attacks by modern NLMPC [20–22].

Figure 19 displays frequency oscillations of the three sources in Scenario 2. Under steady state, all operate near 60 Hz. At t = 35 s, the FDI attack causes measured frequencies to spike unrealistically to nearly 100 Hz, distorting signals and disrupting control. This figure highlights the direct negative effect of FDI attack on the system dynamics and frequency stability.

**Fig. 20:**
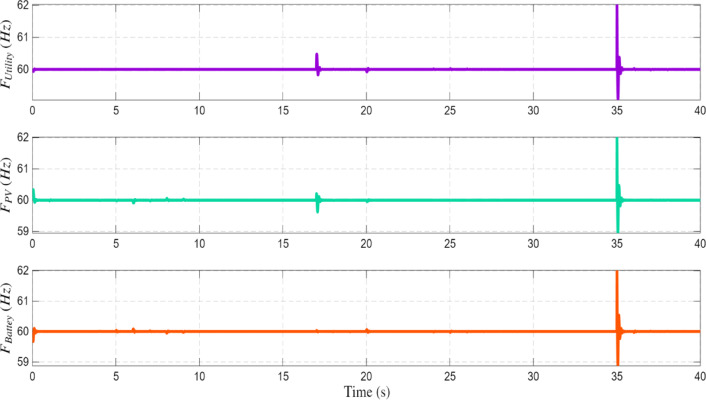
Frequency response of MG under FDI attacks by proposed robust data-driven NLMPC.

Figure 20 provides the frequency behaviour of three inverters in scenario 3. For the first 35 s, frequencies remain near 60 Hz. At t = 35 s, the FDI attack causes a sudden disturbance in the measured frequency signals. However, it is observed that oscillations are rapidly reduced, and the system can restore to a stable state. This behavior confirms that the proposed robust data-driven NLMPC framework can effectively detect and mitigate the impact of the cyberattack while ensuring frequency stability. This figure emphasizes the enhanced resilience and reliability achieved through the proposed control strategy under FDI attack scenarios.

**Fig. 21:**
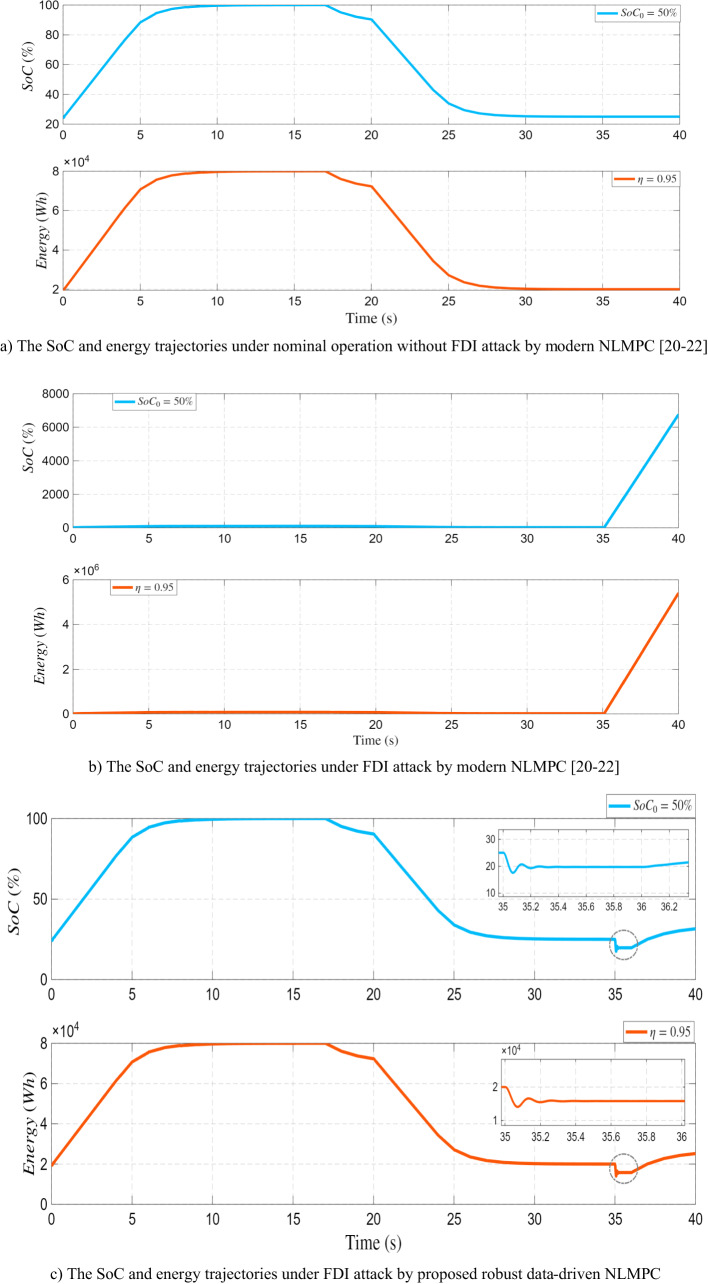
The SoC and energy trajectories under different scenarios.

In Fig. 21a, SoC initially rises from 20% to near 100% within the first 10 s as the BESS absorbs excess energy from the main grid. Between 10 and 20 s, the SoC remains constant, indicating a balance between charging and discharging. After 17 s, the BESS begins to discharge, causing a decrease in SoC and total stored energy. At 20 s, the BESS discharges again to contribute to supplying reactive power to the network. It should be noted that the lower (20%) and upper (100%) SoC limits are intentionally set within the control strategy to prevent deep discharge and overcharge conditions, respectively. These protection limits are implemented to preserve the electrochemical health of the BESS, minimize degradation, and extend its operational life. The symmetrical and smooth charging/discharging profile within these limits further confirms that the modern NLMPC provides effective power management while maintaining BESS longevity and safety under normal conditions. Alternatively, Fig. 21b displays the system response in the case of the FDI attack at t = 35 s. Under this attack, the modern NLMPC loses stability, frequency deviation grows rapidly, and SoC and energy trajectories diverge, displaying unphysical and uncontrolled behavior. This indicates corruption of measurements and misinterpretation of falsified feedback signals, which can lead to overcharging or deep discharge if not addressed. This figure highlights the vulnerability of the modern NLMPC to cyberattack and the potential for catastrophic system failure. Figure 21c shows the same FDI attack on the proposed robust data-driven NLMPC framework. With real-time BNN-based anomaly detection and control re-optimization, the controller identifies unusual deviations and reduces their effects. A minor transient at t = 35 s causes the SoC to momentarily drop below the lower limit (20%) to maintain overall system stability. However, the SoC and energy trajectories remain physically valid and fully recover within0.4 s. Comparing the three cases, Fig. 21a and c exhibit realistic SoC and energy profiles, and Fig. 21b shows full control loss. The proposed robust data-driven NLMPC restores system integrity, keeping safe ranges (20–80% or 20–100%) and ensuring smooth energy management with ramp-rate constraints. The results confirm that integration of BNN-based modeling with probabilistic optimization significantly enhances the robustness, reliability, and cyber-resilience of the MG energy storage system.

### Discussion

The simulation results presented in Table 2 provide a comparative analysis of the suggested robust data-driven NLMPC approach across other operating scenarios. For having a fair baseline comparison, both the traditional modern NLMPC and the proposed BNN-based robust data-driven NLMPC were tested under identical operation including the same load variations, the same renewable uncertainty realization, and same FDI attacks injection time and magnitude. The performance of the two controllers is within an acceptable range for regulation under normal conditions. However, during the same attack on the system, the baseline controller experiences loss of stability with large frequency and power oscillations, while the proposed framework quickly identifies the presence of the attack, re-optimized the control input and restores stable performance. This direct comparison shows that the observed performance improvements arise from the proposed uncertainty-aware detection and reduction mechanism rather than difference in operation scenario. Under normal operation (Scenario 1), the modern NLMPC maintains frequency deviation within ± 0.5 Hz, achieving a settling time of less than 0.3 s, and keeps the SoC within 20–100%. These results confirm that the baseline controller ensures secure battery operation while providing robust dynamics performance under nominal operation conditions. Scenario 2, representing an unprotected FDI attack, indicates that the system becomes very unstable: frequency deviation is 95.6 Hz, while SoC and energy trajectories diverge, causing uncontrollable oscillations in active and reactive power. This outlines the vulnerability of modern NLMPC to cyber-physical attacks and the potential risk of BESS damage and network collapse. Conversely, the proposed robust data-driven NLMPC (Scenario 3) shows significant improvement in all metrics. With a BNN-based anomaly detector and real-time re-optimization, the controller identifies the injected false data within 0.1 s and restores system stability within 0.4 s. Frequency deviation is reduced by approximately 99.7%, active power oscillations by 85%, and reactive power oscillations by 87% compared with Scenario 2. The SoC of the BESS remains within a healthy range of 20–100%, and energy trajectories exhibit smoother behaviour, with roughly 10% improvement. Settling time decreases by around 17% compared with normal operation, indicating improved dynamic performance and smoother system response. These results demonstrate that the proposed framework not only mitigates cyber-physical attacks but also enhances baseline performance, guaranteeing operational reliability, safety, and battery protection. The probabilistic decision-making and adaptive control of the BNN-improved NLMPC provide a comprehensive solution for detection, mitigation, and recovery, effectively addressing the emerging research gap in resilient MG operation under uncertainties and cyber-attacks.

**Table 2:** Comparative performance of modern and robust data-driven NLMPC under different operating scenarios.

Performance metric	Scenario 1 [20–22]	Scenario 2 [20–22]	Scenario 3	Improvement compared to scenario 2	Improvement compared to scenario 1
Frequency deviation (Hz)	± 0.5 (stable)	95.6 (unstable)	± 0.3	99.7% reduction	40% smoother regulation
Settling time (s)	< 0.3	Unstable/divergent	< 0.25	≈ 92% faster recovery	≈ 17% faster
SoC range (%)	20–100 (safe)	Unstable/divergent	20–100 (safe, transient < 0.4 s)	Fully restored to limits	Enhanced protection (regulated to 20–80% window)
Energy range (Wh)	2 × 10^4^ – 8 × 10^4^	Unstable/divergent (> 5 × 10⁶)	2 × 10^4^ – 8 × 10^4^ (stable, smoother dynamics)	Fully restored	~ 10% smoother energy trajectory
Active power oscillations	< 2%	± 80–90%	< 1.5%	85% reduction	~ 25% reduction
Reactive power oscillations	< 3%	± 70–80%	< 2%	87% reduction	~ 33% reduction
Battery protection enforcement	Maintained	Violated (overcharge & deep discharge)	Maintained (20–80% safety band)	100% protection restored	Improved SoC control margin
Reliability/confidence level	90–95%	< 10%	95–98%	Fully restored / > 9 × improvement	~ 3% reliability gain

Compared to the conventional deterministic NLMPC and other data-driven techniques that do not consider uncertainty quantification, the proposed framework exhibits significant performance improvements. For instance, the frequency deviation is reduced by approximately 99.7%, and active and reactive power oscillations are reduced by 85% and 87%, respectively. Moreover, the stability of the system is restored in 0.4 s under FDI attacks, emphasizing the robustness of the proposed framework.

In order to further examine the robustness of the developed framework under uncertainty, a sensitivity analysis is performed using Monte Carlo simulations. Stochastic variations in renewable energy sources, load conditions, and measurement disturbances are propagated in the BNN-based predictive model. It allows the controller to adapt to a wide range of conditions. The results show that the proposed approach keeps its performance under variations, with stable frequency regulation, bounded SoC, and reduced power fluctuations. This indicates the robustness of the proposed framework against both parametric uncertainties and attack-related disturbances. In addition, the probabilistic nature of the BNN allows uncertainty-aware decision-making, where the prediction variance directly influences detection and control adaptation process.

## Conclusion

The paper proposes a robust data-driven nonlinear model predictive control (NLMPC) framework with Bayesian neural networks (BNNs) to enhance cyber-resilient real-time operation of microgrids. The proposed framework performs probabilistic state estimation, anomaly detection, and control reconfiguration within a unified loop, enabling coordinated handling of system nonlinearities, uncertainties, and cyberattacks. Simulation results validate the performance of the proposed framework under different operating conditions. Under FDI attacks, the controller detects anomalies within 0.1 s and restores system stability within 0.4 s. Compared to the unprotected system, the proposed framework reduces the frequency deviation by approximately 99.7%, and the active and reactive power fluctuations reduced by 85% and 87%, respectively. Moreover, the SoC level of the battery is maintained within the safe range of 20–100%, avoiding overcharge and deep discharge, and ensuring reliable energy management. Additionally, the proposed framework improves the system performance under normal operating conditions, reducing settling time by about 17%, and enhancing energy trajectories smoothness by about 10%. The BNN-based uncertainty estimation further increases reliability to 95–98% and enables uncertainty- awareness decision-making, resulting in more robust and adaptive control actions. These findings confirm that the proposed BNN-based NLMPC approach presents a comprehensive solution for cyber-resilient microgrid control, ensuring stability, fast recovery, robustness, and battery health under uncertainties and cyberattacks. They provide valuable insights for developing uncertainty-aware and cyber-resilient control strategies the future smart grid systems.

These results offer actionable insights for developing cyber-resilient and uncertainty-aware microgrid control systems, which highlights the value of integrating probabilistic learning models into real-time predictive control systems.

While this proposed framework achieves real-time performance for investigated microgrid, some limitations should be acknowledged. At first, the computational burden may increase in large microgrids due to the number of states, control variables and uncertainty propagation samples grow with scale, which can make real-time implementing the real-time optimization difficult. The second aspect is that the proposed framework has been verified through simulation and practical implementation in large networked microgrids. It may require model reduction, distributed or multi-agent control structures, and additional hardware verification. In other words, although the current findings prove the feasibility and effectiveness of the proposed approach in the studied microgrid, future studies is needed to enhance scalability and deployments readiness for large practical microgrids.

## Future directions

In future work, the resilient NLMPC will be scaled to larger, practical microgrids, including HIL testing to assess real-time performance under delays, nonlinearities, and noise. Extensions will focus on multi-microgrid systems with coordinated, multi-agent control. Moreover, research will investigate adaptive uncertainty quantification, dynamic FDI detection thresholds, and integration with edge computing and IoT for scalable, autonomous, and attack-resilient operation supporting next-generation smart energy systems.

## Data Availability

The datasets used and/or analysed during the current study available from the corresponding author on reasonable request.

## List of symbols

ANN: Artificial neural network
BNN: Bayesian neural network
BESS: Battery energy storage system
DG: Distributed generation
DiG: Diesel generator
FDI: False data injection
GPR: Gaussian process regression
MG: Microgrid
NLMPC: Nonlinear model predictive control
$\mathcal{u}$: Control input vector
${E}_{\mathfrak{B}ESS}$: Stored energy in BESS
$\mathfrak{Z}$: Disturbance or noise vector
$X$: System state vector
μ: BNN mean prediction
$\mathcal{Y}$: System output vector
$\omega$: Angular frequency (rad/s)
${\mathcal{Y}}_{ref}$: Reference for output trajectory
${\omega }_{0}$: Nominal angular frequency (rad/s)
σ^2^: BNN uncertainty variance
$\mathcal{S}oC$: State of charge
$\Sigma$: Covariance matrix of uncertainty
$$\theta$$: Phase angle
